# Seasonal and diurnal measurement of ambient benzene at a high traffic inflation site in Delhi: Health risk assessment and its possible role in ozone formation pathways

**DOI:** 10.5620/eaht.2023016

**Published:** 2023-08-23

**Authors:** Poonam Kumari, Daya Soni, Shankar G. Aggarwal, Khem Singh

**Affiliations:** 1 CSIR-National Physical Laboratory, Dr. K.S. Krishnan Marg, New Delhi, India; 2 Academy of Scientific and Innovative Research (AcSIR), Ghaziabad, India

**Keywords:** Benzene, GC-FID, risk assessment, ozone formation potential, meteorological parameter

## Abstract

Benzene is the most toxic and hazardous pollutant among volatile organic compounds (VOCs), as it comes under group 1 carcinogens recognized by the International Agency for Research on Cancer (IARC). It also plays a significant role in forming secondary pollutants like ozone. The benzene concentration was measured using a charcoal sorbent tube by active sampling at a traffic junction and analysis was done using GC-FID. The maximum average concentration of benzene in ambient air was found to be 33 μg/m^3^. A diurnal study of benzene measurement shows higher benzene concentrations in the evening compared to the morning. Seasonal variation of benzene is found to be winter > spring > summer > autumn > monsoon and OFP was found to be 21, 19, 14, 13, and 10 respectively. Cancer (ILCR) and non-cancer (HQ) health risk assessment was done to determine the impact of ambient benzene on the residents of urban areas. The yearly average value of ILCR was found to be 2×10^-6^ ± 1×10^-6^ which ranges from acceptable value to three times the WHO acceptable value i.e 1×10^-6^. The correlation of ozone and its precursor, benzene with meteorological parameters is also evaluated. The correlation of benzene and ozone with solar radiation shows the influence of photochemical reactions on the levels of benzene and ozone at the study site, although it is low.

## Introduction

Volatile organic compounds (VOCs) like benzene, toluene, ethylbenzene, and xylenes are hazardous pollutants introduced into ambient air through many natural and anthropogenic activities. Among all of the above, “benzene” is the most carcinogenic pollutant present in ambient air and is a silent killer for the environment as well as humans. Based on human exposure and animal studies, the risk assessment guideline [1] says that benzene is a "known human carcinogen" (Category 1). The National Ambient Air Quality Standard (NAAQS) for 2009 lists it as a pollutant. The annual specified limit for benzene is 5 µ g/m^3^. But there is no short-term exposure standard for benzene. The International Agency for Research on Cancer (IARC) [2] says that benzene is a dangerous and toxic pollutant that can cause cancer and is a group 1 carcinogen. There are many human health effects due to low and high exposures to benzene. Long-term exposure to benzene concentration in ambient air may cause aplastic anaemia, respiratory diseases, chronic myeloid leukaemia, etc. in human beings [3-12]. Many studies have observed that in pregnant women, exposure to benzene or other organic solvents may increase the chance of low birth and fetal malformations and have an effect on baby weight growth [13-15]. Benzene exposure can cause skin and eye irritation, problems in the throat (coughing and wheezing), headaches, dizziness, vomiting, nausea, coma, and even sudden death. Long-term effects of benzene include many kinds of cancer, and leukaemia can happen if you are exposed to it for months or years. It might make them less able to have children and more likely to have chromosomally damaged sperm, which can cause unplanned abortions, mental problems, and problems that are passed down to their children [16]. Naturally, it is emitted into the ambient air by volcanoes as they become active and ejects lava and gases, from forest fires, and is found in some plants. It is evaporating into the air rapidly, and its vapor, which is heavier than air, may dip below ground level. After contact with soil, it can break quickly and may contaminate groundwater. Sources made by people include gasoline refineries, the evaporation of fuel during loading and unloading, storage tanks, industry and chemical solvents used in industry and household products, vehicle exhaust from both complete and incomplete combustion of gasoline, cigarettes, paints, etc. About 99.3 % of all VOCs in the air come from the transportation sector [17]. A. Kumar et al. [18] found that between 80 to 85 % of vehicle exhaust, 10 to 20 % of vaporization, and 3 to 6 % of benzene are released by the transportation sector.

Benzene plays a major role in the formation of secondary pollutants like ozone [18]. In the presence of sunlight and hydroxyl radicals (OH), benzene and its substitute take part in photochemical reactions that create SOA and peroxyacetyl nitrate (PAN) [19-21]. Most tropospheric ozone is made when VOCs, nitrogen dioxide, and OH radicals interact with light [22-23]. Due to the oxidation of VOC, the secondary organic aerosol is made in the atmosphere, which has a big effect on particulate matter [24]. Ground-level ozone can impact human health [25] and vegetation [26]. Many studies also point out that benzene, as a precursor to the formation of atmospheric (SOA) secondary organic aerosol [27-30] has the largest OFP (ozone formation potential) contributions [31].

The present study is of great concern not only because benzene can cause carcinogenic effects in humans and animals but also because it is an important precursor of ground-level ozone. This study focuses on determining the concentrations of benzene at urban area traffic junction for the years 2021–2022, health risk assessment from ambient benzene exposure, and the chemical reactivity of benzene species to the formation of ozone as measured by ozone formation potential. The results were also compared with the online monitoring data taken for the same site and the same duration. Metrological parameters such as Temperature, Humidity, and Wind speed & direction, and Solar radiation are strongly related to ozone formation and benzene concentration in ambient air. A correlation between them is also established on the basis of measured benzene concentration and OFP. This study is providing reliable data to help the regulatory bodies for applying strategies for pollution measurement.

## Methods

### Study area

**Figure 1.**
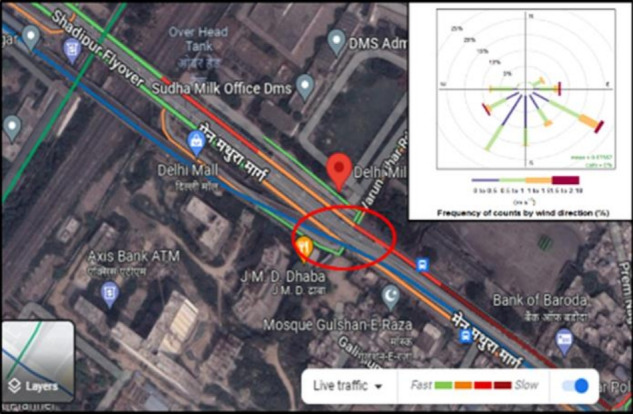
Location map of sampling point, Shadipur, New Delhi

Delhi has been selected for the benzene monitoring, which is in northern India and spread over an area of 1483 km2 between the latitudes of 28°-53’-00” and 28°-24’-17” North and the longitudes of 77°-20’-37” and 76°-50’-24” East. Haryana and Uttar Pradesh are the two border states of Delhi. Due to the tropical wet and dry climate, very low and very high temperatures are observed. It is very cool in the winter and hot in the summer. The climate of Delhi is split mainly into five seasons: December to January (winter), February to March (spring), April to June (summer), July to Mid-September (monsoon), and End September to November (autumn). During the winter season, the average temperature in Delhi can vary from 22 °C to 5 °C, and during the summer season, it can vary from 25 °C to 45 °C. The wind flow direction is generally from west to northwest, but on rainy days it changes to south-southwest. The samples were taken at Shadipur Fig.1 in west Delhi, close to a commercial and residential area. It is near a high-traffic junction connecting to the main Mathura Marg.

### Sampling

Sampling was done from September 2021 to August 2022 in alternate weeks during rush hours (8–9 AM) with high traffic movement. For the diurnal study, sampling was done two times a day (morning and evening) in the summer and winter months (twice each month). Air was sucked through a battery-operated low-flow sampler (Enviro Tech; APM 800), and to control the flow rotameter, it was calibrated at CSIR-NPL. Air sampling was done at 100 mL/min for 60 minutes using a charcoal sorbent tube. The sorbent tubes (Coconut charcoal, SKC, Anasorb CSC, 6×70-mm size, two sections, 50/100- mg sorbent) were used for air sampling. Sampling was done approx. 1-1.5 m above from ground in a scheduled manner and charcoal tube hold vertically to avoid breakthrough. After sampling, the tube ends were closed with a cap and wrapped with aluminium foil. Then the analysis was done using Method NIOSH 1501 (National Institute for Occupational Safety and Health) within 1–2 hours by gas chromatography using a flame ionization detector (Agilent 6890N). Details of the sampling procedure are shown in Figure S1. During the study period, a real-time online monitoring station was used to measure the meteorological parameters to see how it affected the amount of benzene in the ambient air.

### Reagents

Pure benzene > 99.9 % purity, purchased from Sigma Aldrich, was used for the making of calibration standards, and HPLC-grade acetone was used as the solvent for extraction.

### Analysis

Analysis was done using a gas chromatograph (Agilent), Model 6890N, using a HP-5 (5% Phenyl methyl siloxane) capillary column with 30 m length × 0.32 mm ID × 0.25 μm thick film, the initial temperature was maintained at 50 °C and held for 2 min, raised to 100 °C at a rate of 20 °C min^−1^, then raised to 150 °C at a rate of 30 °C min^−1^ and held for 2 min and again raised up to 180 °C at a rate of 40 °C min^−1^ and held for 8 min. Injector temperature is maintained at 200 °C with split mode (1:5), using as carrier gas nitrogen at a rate of 1 mL min^−1^, detector temperature was kept at 250 °C, with air at 400 mL min^−1^ and hydrogen at 60 mL min^−1^. A four-point calibration curve was obtained for benzene by preparing a standard solution of benzene in acetone with a concentration of 0.5 ppm to 5 ppm. The linear correlation coefficient value was obtained at 0.99. A chromatogram of benzene is shown in figure S2, showing the detector response for the calibration standard solution of benzene and an unknown air sample of benzene.

### Health risk assessment methodology

Cancer and non-cancer health risk assessment were performed to determine the impact of ambient benzene pollutants through the inhalation pathway. The impact was estimated by using the incremental lifetime cancer risk (ILCR) and hazard quotient (HQ) equations for health risk assessment that is recommended by the United States Environmental Protection Agency (USEPA) guidelines [32] and the Integrated Risk Information System (IRIS). The HQ equation was used to figure out the non-cancer health risks of this pollutant, while the ILCR was used to figure out the cancer risks. The lifetime average daily dose (LADD) was calculated to find out the ILCR by considering the exposure through the inhalation pathway.

The ILCR was calculated using Eq. (1). Where LADD is the lifetime Average Daily Dose (mg/kg-day) and CSF is the cancer slope factor (mg/kg-day) of benzene. The CSF of benzene (0.0273 mg/kg-day) is given in the Risk Assessment Information System (RAIS) [33].


(1) ILCR=LADD×CSF

The LADD of employees is calculated using Eq. (2)


(2) $$LADD=\frac{CA\times CF\times IR\times EF\times ET\times ED}{BW\times AT}$$

where,

CA (μg/m^3^) = Contaminant Concentration in Air

CF = Conversion Factor (1 mg/1000 μg) i.e., 0.001 mg/μg

IR (m^3^/h) = Inhalation Rate as per US EPA standard (20 m^3^/day) i.e., 0.83 m^3^/h

ET (h/day) = Exposure Time (Consider 24 h/day)

EF (days/years) = Exposure Frequency (365 days/years)

ED (years) = Exposure Duration (1 year)

BW (kg) = Body Weight (70 kg, average body weight) (US EPA standard)

AT (day) = Averaging Time (70 years× 365days/years)

CSF (mg/kg-day) = Inhalation Cancer Slope Factor

All the values for the risk assessment parameters used in equation (2) are given in Table S1. Because sampling of benzene was done for 1 hrs, but above all calculation done considering continuous exposure (24 h/day) same month wise observed concentration of benzene till one year. So obtained result is overestimated for residential area.

The ILCR value ˃1×10^-6^ was considered to have carcinogenic effects of concern, and a value ≤ 10^-6^ was considered an acceptable level [34]. Risk assessment for non-cancer risk was expressed by the HQ value, calculated according to the following equation:


(3) $$HQ=\frac{LADD}{RfD}$$

The RfD value for benzene is 0.03 mg/kg-day [35].

The value of HQ ˃ 1 indicates adverse non-carcinogenic effects of concern, and the value of HQ ≤1 was considered an acceptable level [34].

### Ozone Formation Potential (OFP)

The MIR method is used to determine the OFP of individual VOCs, as developed by Carter [36]. The updated MIR constant [37] is used in this study for the calculation of OFP, which was calculated using Eq. (4). where OFPi is the ozone formation potential of chemical i (here, i = benzene). MIRi is the maximum incremental reactivity scale of chemical ‘i’ and Ci is the ambient concentration of chemical ‘i’.


(4) $${\mathrm{OFP}}_{i}={\mathrm{MIR}}_{i}\times C_{i}$$

Generally, MIR values are used to determine the relative tropospheric ozone impacts of VOCs. MIR popular unit coefficients were designed by Carter to be used in relatively high NOx conditions to determine the OFP of different VOC compounds.

### Chemistry of benzene towards the formation of secondary pollutants

Benzene being a VOC plays a big role as a precursor in the formation of ozone and other secondary pollutants in ambient air. Secondary Organic Aerosol (SOA) formed from photo-oxidation of benzene. Ground-level ozone, peroxyacetyl nitrates, peroxides, and other chemicals are produced when sunlight hits VOCs and nitrogen oxides (NOx). This process is called photo-oxidation. VOCs are released from natural (plants) and anthropogenic sources such as vehicle exhaust, consumer products, evaporating fuels and solvents etc. More than 85 % of the NOx that is put into the air comes from power plants, industry, and the burning of fossil fuels [38]. The presence of NOx and VOCs in the environment is contributing to the formation of ozone [21].

In the case of benzene, the OH radical addition reaction proceeds when H-atoms are abstraction from the C-H bonds of the aromatic ring. This makes a hydroxycyclohexadienyl, also called an OH-aromatic adduct. A detailed mechanism of reaction occurring during formation of Secondary Organic Aerosol (SOA) and ozone from benzene described in literature [21]. The overall reaction between VOC and NOx (primary pollutants) toward the formation of secondary pollutants (troposphere ozone and PAN) is given as


(5) $$\mathrm{VOC}+{\mathrm{NO}}_{X}+\mathrm{h\vartheta}\to \begin{array}{c} O_{3}\\ \mathrm{Ozone} \end{array}+\begin{array}{c} \mathrm{PAN}\\ \mathrm{Peroxyacetyl}\;\mathrm{nitrate} \end{array}\;$$

## Results and Discussion

### Benzene concentration

**Figure 2.**
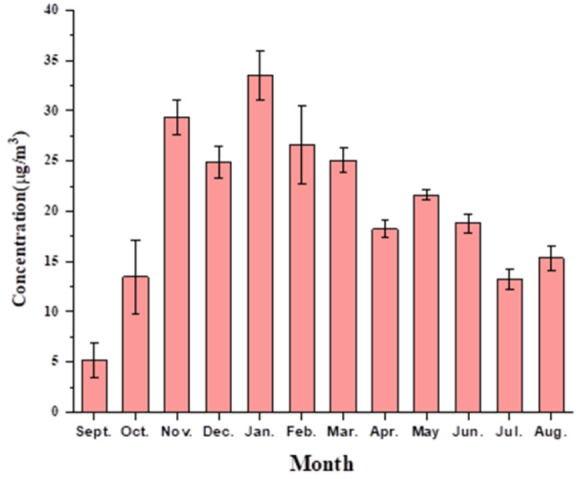
Monthly average concentration of benzene in ambient air at Shadipur

The level of benzene in ambient air at Shadipur was observed for one year, from September 2021 to August 2022. A detailed monthly concentration range of benzene with uncertainty is given in Table S2. Figure 2 is graph that shows the average amount of benzene in the ambient air each month. In January, maximum concentrations of benzene were observed in the range of 24-43 μg/m³. Minimum concentration was observed in September in the range of 2–6 μg/m³ i.e., the period after the end of the monsoon. During the overall study, the concentration of benzene was observed to be much greater than the annual prescribed limit, i.e., 5 μg/m³ as per the NAAQS 2009 of the Central Pollution Control Board, New Delhi. The high concentration of benzene at the study point may be due to the high automobile traffic density [39] the slow movement of vehicles, and the constant idling of the vehicles during the sampling period. The transportation sector is the major source of benzene emissions, which are reported at 80–85 % in ambient air [18]. Aside from vehicle emissions, other anthropogenic sources of pollution can include factories, construction sites, gas stations for cars, electric motor winding, etc. In colder months, December to February, maximum mean concentration of benzene concentration was obtained in January month while the minimum concentration was observed in December month supported by the same pattern in literature [40]. High level of benzene in these months can be attributed to slow photochemical reactions due to lesser intensity of solar radiation and calm atmospheric conditions.

Observed data of benzene in ambient air from the online monitoring station (1-hour average) for the same period of sampling done by adsorption on a charcoal tube are shown in figure S3. The concentration of benzene observed exceeded the NAAQS limit of 5 μg/m^3^. This may be attributed to the sampling height, as the online monitoring data was taken for the air samples at a height of approximately 3 m, but for the manual sampling, the samples were collected at the height of a person's breathing zone (approximately 1-1.5 m from the ground).

### Temporal variability of benzene

**Figure 3.**
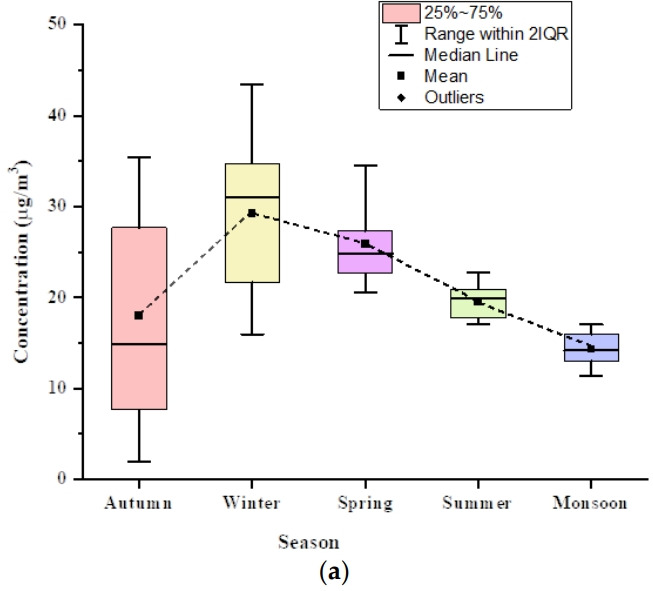
Boxplots represent the total benzene concentration variation in different seasons. The square inside the rectangular box shows the average concentration of benzene. The box shows the interquartile range, with the 25^th^ percentile (the first quartile) and the 75^th^ percentile (the third quartile) at the top and bottom of the box, respectively. The upper side of the whisker denotes the maximum value, and its lower side corresponds to the minimum value.

Fig. 3 illustrates the variability of benzene mean concentrations in different seasons. The trend of benzene concentration in ambient outdoor air is found to be winter > spring > summer > autumn > monsoon. Although, traffic inflation is reasonably constant throughout the study period (morning office hours), the higher level of benzene in winter season might be due to the calm condition and stability of atmosphere due to stable meteorological parameters. The higher range of benzene in the winter may be caused by lesser photochemical reactions because the sun is not shining as brightly, a low rate of dispersion, a lower mixing height, no dilution of pollutants because of temperature inversion, and stable atmospheric conditions [41]. The level of benzene was observed to be highest in the winter season, which is also supported by other literature studies [39,41,42]. A detail of benzene concentrations (μg/m^3^) measurement with the other similar studies is shown in Table S3.

Benzene concentration was found to be lower in the summer and spring seasons than in the winter, and a similar trend was found in other studies [43-45]. Benzene was found to be at its lowest level during the monsoon season. This is because rain washes away pollutants in the air and lowers the amount of benzene in the ambient air. In the summer, on the other hand, atmospheric dispersion is more important because the boundary layer is deeper, there is more sunlight, and temperatures are higher, all of which speed up photochemical reactions. Hence, the removal of benzene by OH radicals in the summer is greater than in the winter [41,46]. In summer, the concentration of OH radicals are high due to the photolysis of chemical species like aldehydes, ozone, and terpenes. The production of OH radicals could also increase from the reaction of terpenes with O3 [47]. Similar seasonal concentration trends for benzene were observed in Delhi [39, 48]. The level of benzene during the summer and monsoon seasons was relatively low as compared to the winter season, as supported by the literature [49]. The mean benzene concentration in the autumn season is higher as compared to the monsoon and lower than that in the summer. This may be attributed to the localized factors that are the result of the festive season during the autumn. Changes in the amount of benzene over time showed how weather conditions affected the amount of the pollutant.

The higher concentration of benzene in winter than summer month was further validated by applying student t-test assuming unequal variance for two samples. The null hypothesis indicates that the mean concentration of benzene for both seasons (winter and summer) is the same. P-value and t-value were found to be 0.00008 and 4.831. The result shows that t test is found to be significant (p<0.05) with 95 % confidence level and null hypothesis is rejected at 5 % confidence level. Hence, it can be further concluded that there is a difference between the mean value of benzene for both the winter and summer seasons.

### Health risk assessment

In this study, the estimated cancer health risk assessment of benzene was calculated using Eqs. (1) and (2), and the non-cancer health effect of benzene was calculated as per Eq. (3). Table 1 shows the value of monthly and yearly average lifetime cancer (ILCR) and non-cancer risk (HQ) calculated for benzene, considering 24 hrs per day continuous exposure (as observed from 1 hrs sampling at traffic junction) of benzene in ambient air.

During the study period, the average cancer risk and non-cancer risk associated to benzene in the air were measured each month. The ILCR value ≤ 10^-6^ was considered acceptable according to WHO [34]. The ILCR value for a monthly average of benzene in the air exceeded the values given by the WHO and the USEPA. We observed from Table 1 that the HQ value was lower than the WHO limit of 1 during the overall study hence there is no risk associated with noncancer health effects. Yearly average ILCR value ranges from 0.6 × 10^-6^ to 4 × 10^-6^ and given in Table 1 with standard deviation. The estimated value of cancer risk is having almost 50% standard deviation which expressed the reliability and overestimation (if any) of the data. In month of September, October and July, the ILCR value is as per WHO acceptable range (ILCR ≤ 10^-6^), but in other months it exceeded the limit.

### Ozone formation potential assessment

VOCs play a significant role in the photochemical production of ozone. The OFP is used as an index to estimate the potential of VOC compounds in ozone production and to estimate the impact of individual components in the process of ozone formation. In this study, season-wise OFP for benzene is calculated from equation 4 using an updated MIR coefficient of 0.72 [37,50] given in Table S4. The reported result of OFP is found to be less than that reported by other studies in New Delhi [39,51]. The OFP has been determined using the MIR coefficients in the Carter equation in different studies for specific VOCs toward ozone formation [19,39, 52] but all these studies used older MIR coefficients in their OFP calculations.

**Figure 4.**
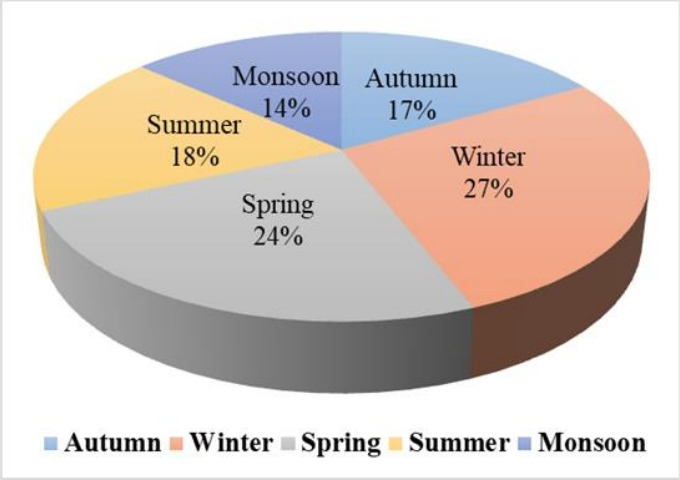
Seasonal OFP contribution of benzene in percentage

Figure 4 shows that ozone formation potential was observed to be maximum in the winter season (27 %) and minimum during the monsoon season (14 %), of the total OFP, during the study period. The seasonal OFP trend is the same as for the concentration of benzene, i.e., winter > spring > summer > autumn > monsoon. Season-wise ozone formation potential is reported as winter > autumn > summer > spring [27] for VOC, with benzene contributing 31% of the total formation potential of secondary organic aerosol. According to the most recent research benzene from metal packing plants is the main source of OFP, making up 65% of the total OFP [31]. Metrological parameter such as temperature, solar radiation, and wind speed strongly impact on ozone formation potential. The maximum OFP value was observed in the winter season in this study, which is supported by the literature [27].

### Correlation of benzene concentration with meteorological parameters

Meteorological parameters (temperature; wind speed & direction; relative humidity and solar radiation) were recorded (24 hourly average) during the study period from the online air quality monitoring station at Shadipur. To figure out what the relationship is between the benzene concentrations and the weather variables, the correlation coefficients have been calculated and are shown in Table 2. The strength of the relationships was measured by the size of the r-values. The results showed that the amount of benzene is inversely related to both temperature (-0.73) and wind speed (-0.34) but was found to be non-significant (at α=0.05) with temperature and significant with wind speed (at α=0.05).

There was a weak relationship between benzene species and meteorological parameters. It means that the concentration of benzene in the air doesn't depend on the weather at the sampling site. A negative correlation (-0.73) was observed between temperature and ambient benzene. But there is insufficient evidence to conclude that there is a significant linear relationship between temperature and ambient benzene at α=0.05. As temperatures increase, air pollutants move vertically, dilute rapidly, and are blown away by upper-level winds. Table 2 shows that there is positive correlation (0.24) between the amount of benzene and humidity which is significant at α=0.05. Also, there was weak correlation (-0.02) of benzene with solar radiation and positive weak correlation with ozone (0.08) but both were found to be significant (at α=0.05). The metrological parameters have a weak relationship with each other.

**Figure 5.**
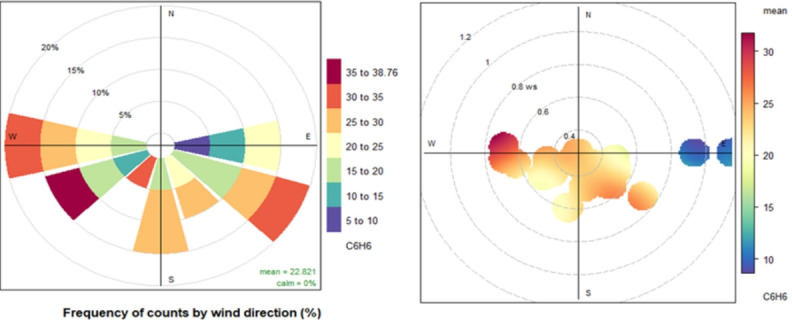
Pollution rose in the graph for benzene (μg/m^3^) with wind speed and direction

From the pollution rose plot in Figure 5, it is seen that higher benzene concentrations are observed when wind speed is slow and blows from a SE-SW direction. As wind speed decreases, the concentration of pollutants is high, whereas as wind speed increases, the concentration becomes low due to dispersion. Maximum concentration on the west side may be due to industrial or construction areas on that side, which may be one of the potential sources.

A weak negative correlation of benzene with solar radiation and a weak positive correlation of ozone with solar radiation show that photochemical reactions are influencing the levels of benzene and ozone at the study site, although they are low. In this study at Shadipur, the same relationship between a metrological parameter and benzene was seen for both online data and data collected by hand.

### Diurnal variation

**Figure 6.**
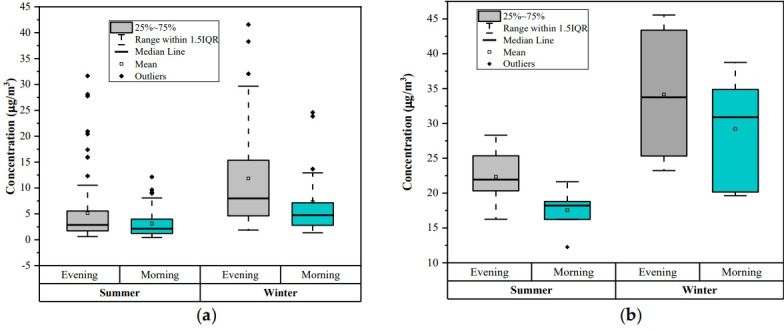
The box plot represents the benzene concentration variation twice a day (morning and evening). While the box distributes interquartile range, the top and bottom of the box are defined as the 25^th^ (the first quartile) and the 75^th^ (the third quartile) percentile, respectively. The upper side of the whisker denotes the maximum value, and its lower side corresponds to the minimum value.: (a) Online monitoring data; (b) Manual sampling data

Diurnal variations of the benzene analysis were done two times in a day, choosing morning time (7 to 8 AM) and evening time (7 to 8 PM) for the two seasons (winter and summer). The diurnal trend for online monitoring data and experimentally measured data at the study location are shown in Figs. 6a and 6b, respectively. Online data was taken from the online monitoring station at Shadipur During the traffic rush hours (morning and evening time) sources of benzene emission is increase and due to low temperature and atmospheric boundary layer height benzene is not spread thus the mass concentrations of benzene is generally high at evening and morning.

As indicated in Fig. 6, concentration of benzene was observed more in winter season than in summer season. Benzene concentration is higher in the evening time than the morning time by both online as well as manual sampling analysis. Benzene level is lower in the morning than evening could be attributed to the atmospheric instability with increasing temperature, breaking the inversion, photochemical reaction intensity and vertical motion of ambient air that can dilute benzene [52]. There was a high peak of benzene observed in the evening. Besides photochemical reactions, the evening traffic rush hour in this city may be another reason for it. In other words, benzene is not easily diluted in the evening because of decreasing temperatures, atmospheric stability, low wind speeds, and night-time inversions. The concentration of benzene observed was higher than 5 μg/m^3^ as prescribed by the NAAQS [53].

## Conclusions

Diurnal and seasonal variations of ambient benzene at traffic junction of urban site was observed, implying vehicular emission is one of the major sources of benzene. Monitoring of benzene in ambient air is important because of its carcinogenic effect on humans and because it is one of the precursors to ground-level ozone. The concentration of benzene was found to be greater than the prescribed annual limit, i.e., 5 μg/m^3^ in ambient air. The minimum monthly average concentration of benzene was 5 ± 2 μg/m^3^ and maximum 33 ± 2 μg/m^3^. The seasonal trend of benzene concentration is found to be in order winter (29 μg/m^3^) > spring (26 μg/m^3^) > summer (20 μg/m^3^) > autumn (18 μg/m^3^) > monsoon (14 μg/m^3^), which is the same for ozone formation potential i.e 21, 19, 14, 13, and 10 respectively. The carcinogenic health risks associated with exposure of benzene were found to be above the acceptable value with 50% variation in the season of winter spring and summer for residential area. But in the season of monsoon and autumn, the ILCR values are within the acceptable limit with 50% standard deviation. Also in the whole year data showed that there is no health risk associated with the non-cancer health risk due the exposure of benzene concentration in the residential area. The study also helps people to understand the estimated values due to exposure of benzene, associated with health risk, affects the formation of SOA in the atmosphere and how the chemical reactivity of benzene species affects the formation of ozone, which is shown by its ozone formation potential values. The results here show that the amount of benzene in the air changes throughout the day in the same way for both manual sampling data and online monitoring station data. The concentration of benzene and the formation of ozone in the air are also influenced by meteorological factors like temperature, wind direction, humidity, wind speed, and solar radiation. The correlation of solar radiation with benzene and ozone shows the influence of photochemical reactions on the level of benzene and ozone at the study site, although it is low.

## Figures and Tables

**Table 1.** ILCR and HQ value associated with the exposure to ambient benzene

Month	Cancer risk (ILCR)^1^	Non-cancer risk (HQ)
September	0.6 × 10^-6^	0.0006
October	1 × 10^-6^	0.002
November	3 × 10^-6^	0.004
December	3 × 10^-6^	0.003
January	4 × 10^-6^	0.004
February	3 × 10^-6^	0.004
March	3 × 10^-6^	0.003
April	2 × 10^-6^	0.002
May	2 × 10^-6^	0.003
June	2 × 10^-6^	0.002
July	1 × 10^-6^	0.002
August	2 × 10^-6^	0.002
Yearly Average^2^	2 × 10^-6^ ± 1 × 10^-6^	0.002 ± 0.001

1 Values are calculated from the average concentration of all month’s study and rounded off to next significant figure

2 Yearly Average value is given with standard deviation.

**Table 2.** Pearson correlation coefficient matrix of benzene with meteorological parameters

	NOx	Ozone	Temp	RH	WS	SR	Benzene
NOx	1						
Ozone	-0.34	1					
Temp	-0.55	0.65	1				
RH	0.18	-0.35	-0.59	1			
WS	-0.29	0.18	0.32	0.15	1		
SR	-0.48	0.08	0.24	-0.34	-0.14	1	
Benzene	0.32	-0.44	-0.73	0.24	-0.34	-0.02	1
Benzene^*^	0.28	-0.42	-0.58	0.14	-0.38	0.003	1

* Correlation with online ambient benzene monitoring station data (24 hourly average) at the same study site at approx. 3 m vertical height.
