# Supplementary material for: Seasonal and diurnal measurement of ambient benzene at a high traffic inflation site in Delhi: Health risk assessment and its possible role in ozone formation pathways
Source: Environ Anal Health Toxicol. 2023 Aug 23;38(3):e2023016. doi: 10.5620/eaht.2023016 (PMC10613561; doi:10.5620/eaht.2023016)
Supplement: Supplementary file 1 [file eaht-38-3-e2023016-Supplementary.docx]

**Supplementary Information**

**Seasonal and Diurnal Measurement of Ambient Benzene at High Traffic Inflation Site in Delhi: Health Risk Assessment and its Possible Role in Ozone Formation Pathways**

**Poonam Kumari^1,2^, Daya Soni^1,2*^, Shankar G Aggarwal^1,2^and Khem singh^1^**

^1^*CSIR- National Physical Laboratory, Dr. K.S. Krishnan Marg, New Delhi-110012, India*

^2^*Academy of Scientific and Innovative Research (AcSIR), Ghaziabad-201002, India*

**Content**

**Figure S1.**  Flow chart for active sampling of benzene in ambient air.

**Figure S2.** Gas chromatograph showing peaks of different concentration of benzene

**Figure S3.** Benzene concentration from manual sampling and online monitoring station

**Table S1.** Risk assessment parameters used in this study

**Table S2.** Measurement data of benzene at Shadipur area under study

**Table S3.** Detail of benzene concentrations (μg/m^3^) measurement with the other similar studies.

**Table S4.** Season-wise Ozone Formation Potential of benzene

*Corresponding Author: Tel: 91 11 47091628

Email: [dsoni@nplindia.org](mailto:dsoni@nplindia.org)

S1


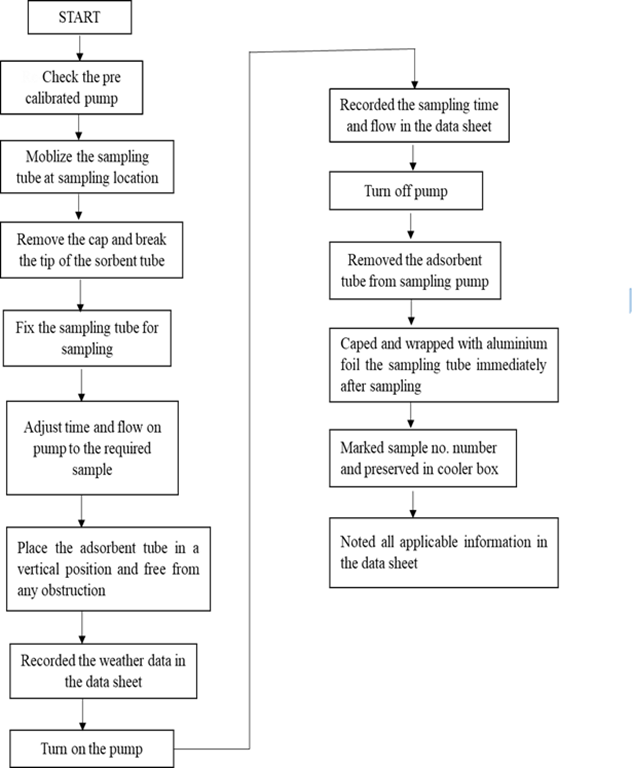


**Figure S1.**  Flow chart for active sampling of benzene in ambient air.

S2


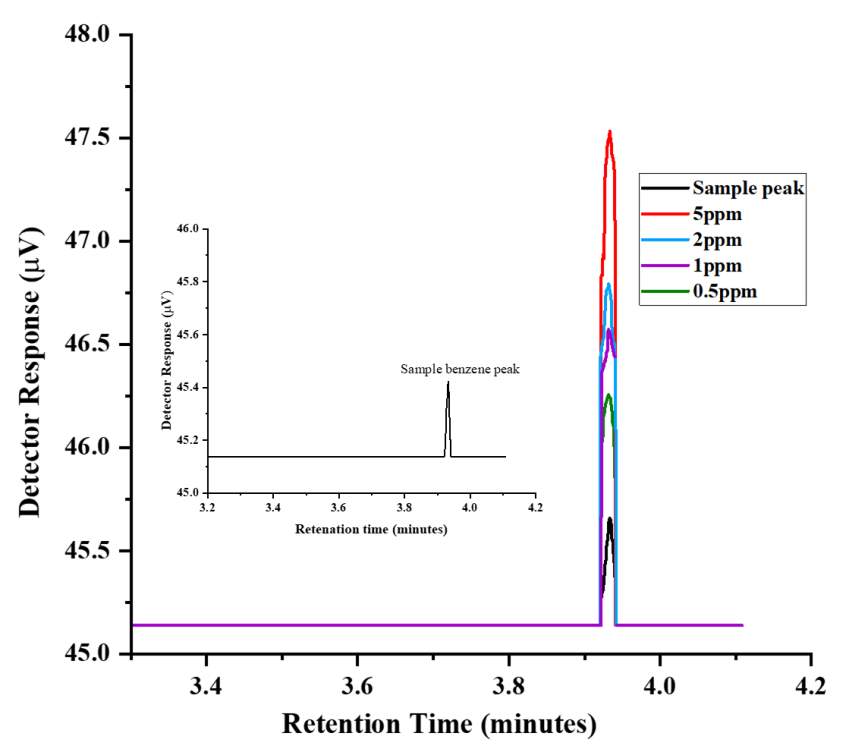


**Figure S2.** Gas chromatograph showing peaks of different concentration of benzene

**Figure S3.** Benzene concentration from manual sampling and online monitoring station

S3

**Table S1.** Risk assessment parameters used in this study

| Parameter | Value* | Unit |
| --- | --- | --- |
| Inhalation rate, adult (IR) | 0.83 | m^3^/h |
| Exposure time (ET) | 24 | h/day |
| Body weight, adult (BW) | 70 | kg |
| Exposure frequency (EF)  Exposure duration (ED) | 365    1 | day/year  year |
| Average lifetime | 70 | year |
| Slope factor | Benzene = 0.0273 | mg/kg-day |

* ^(Garg & Gupta, 2019)^

**Table S2.** Measurement data of benzene at Shadipur area under study

| Month | Benzene (µg/m^3^) | |
| --- | --- | --- |
|  | Range | Average ± u* |
| September 2021 | 2-6 | 5 ± 2 |
| October 2021 | 7-26 | 13 ± 4 |
| November 2021 | 23-35 | 29 ± 2 |
| December 2021 | 16-37 | 25 ± 2 |
| January 2022 | 24-43 | 33 ± 2 |
| February 2022 | 21-34 | 27 ± 4 |
| March 2022 | 22-28 | 25 ± 1 |
| April 2022 | 17-20 | 18 ± 1 |
| May 2022 | 21-23 | 22 ± 1 |
| June 2022 | 17-21 | 19 ± 1 |
| July 2022 | 13-15 | 13 ± 1 |
| August 2022 | 13-17 | 15 ± 1 |

^*u represent Type A uncertainty at^ *^k^*^=1^

S4

**Table S3.** Detail of benzene concentrations (μg/m^3^) measurement with the other similar studies.

| **City** | **Benzene**  **(μg/m^3)^** | **Study area** | **Sampling method/ Measurement Technique** | **Reference** |
| --- | --- | --- | --- | --- |
| Delhi (India) | 2 - 43 | Sadipur, Traffic junction | Coconut charcoal sorbent tubes (SKC, Anasorb CSC, sorbent; extraction with acetone/ GC-FID; Agilent 6890N | This study |
| Delhi (India) | 142.2 | IIT Delhi & UP Boarder, Traffic junction | ORBO™-32 Charcoal tube/  GC-FID; Nucon 5700 gas chromatography | 51 |
| Delhi (India) | 1.98–10.26 | Shahdara, Traffic-Congested Area | Charcoal tubes (ORBOTM32)/ GC-FID | 52 |
| Leon, Guanajuato, Mexico | 1.96 | Urban, Industrial, Heavy traffic area | Active sampling using Charcoal sorbent tubes; 1.5 h; 200 mL min^−1^ ; CS_2_ desorption/ GC-FID | 54 |
| Kolkata,  India | 24.97–79.18 | Urban, Industrial, Heavy traffic area | Active sampling using Charcoal sorbent tubes; 100 mL min^−1^ ; 6 h; CS_2_ desorption/ GC-FID | 55 |
| Iran | 2-108 | Industrial and local road traffic area | Active sampling using activated carbon ,2 h, Sampling pump (SKC Inc., England, model 222-3) | 23 |
| Arad City, Romania | 18.00 ± 1.32 | High-density traffic area | Stainless steel tubes, SKC 1003, SKC Inc., Houston, TX, USA), GC-MS coupled with a thermal desorption system. | 42 |
| Delhi (India) | JNU-48,  Connaught Place-97,  Okhla-89,  AIIMS-110 | Traffic area, industrial area | Diffusive sampling using coconut shell charcoal, Thomas Baker Pvt. Ltd. for one week.  CS_2_ desorption/GC-FID (PerkinElmer Auto-XL) | 39 |

S5

**Table S4.** Season-wise Ozone Formation Potential of benzene

| Season | Concentration (µg/m^3^) | MIR | OFP |
| --- | --- | --- | --- |
| Autumn | 18 | 0.72 | 13 |
| Winter | 29 | 0.72 | 21 |
| Spring | 26 | 0.72 | 19 |
| Summer | 20 | 0.72 | 14 |
| Monsoon | 14 | 0.72 | 10 |
| Yearly | 107 | 0.72 | 77 |

S6
